# Effect of mechanically stimulated saliva on initial human dental biofilm formation

**DOI:** 10.1038/s41598-019-48211-3

**Published:** 2019-08-14

**Authors:** Taichi Inui, Robert J. Palmer, Nehal Shah, Wei Li, John O. Cisar, Christine D. Wu

**Affiliations:** 1 Mars-Wrigley Confectionery, Chicago, IL 60642 USA; 2 National Institute of Dental and Craniofacial Research, National Institutes of Health, Bethesda, MD 20892 USA; 3 Department of Pediatric Dentistry, College of Dentistry, University of Illinois at Chicago, Chicago, IL 60612 USA

**Keywords:** Biofilms, Microbiome

## Abstract

This study evaluated the impact of mechanically stimulated saliva on initial bacterial colonization. Interaction between oral bacteria and both unstimulated and stimulated saliva was examined *in vitro* by laying labeled bacteria over SDS-PAGE-separated salivary proteins. The effects of chewing on *in vivo* biofilm, microbial composition, and spatial arrangement were examined in two human volunteers using an intraoral stent containing retrievable enamel chips. *In vitro* experiments showed that bacterial binding to proteins from stimulated saliva was lower than that to proteins from unstimulated saliva. Lack of binding activity was noted with *Streptococcus mutans* and *Lactobacillus casei*. Human Oral Microbe Identification Microarray (HOMIM) analyses revealed a consistent chewing-related increase in the binding of *Streptococcus anginosus* and *Streptococcus gordonii*. Immunofluorescence microscopy demonstrated the presence of multi-species colonies and cells bearing different serotypes of the coaggregation-mediating streptococcal cell-surface receptor polysaccharides (RPS). Differences in bacterial colonization were noted between the two volunteers, while the type 4 RPS-reactive serotype was absent in one volunteer. Cells reacting with antibody against *Rothia* or *Haemophilus* were prominent in the early biofilm. While analysis of the data obtained demonstrated inter-individual variations in both *in vitro* and *in vivo* bacterial binding patterns, stimulating saliva with multiple orosensory stimuli may modulate oral bacterial colonization of tooth surfaces.

## Introduction

Supragingival biofilm formation is widely recognized as a step-wise process in which acquired enamel pellicle is a specific binding target of early-colonizing bacteria; late-colonizing bacteria are recruited in part by cell-cell recognition called coaggregation and coadhesion^1^. Nascent supragingival biofilms can be detected as early as 2 hours (molecular approach) or 4 hours (microscopy) on clean enamel surfaces^2,3^. The daily oral hygiene regimen of toothbrushing creates a cycle of plaque removal and re-colonization. Therefore, it is important to understand the mechanism of dental biofilm formation at its initial stage as well as to create efficient plaque removal strategies. Recently, Hertel *et al*. used fluorescent *in situ* hybridization to investigate initial oral biofilm colonization patterns of selected oral bacteria from children whose DMFT scores varied^4^. No statistically significant differences in distribution patterns of the adherent bacteria were seen between or among the DMFT groups, although the low-DMFT group showed the least amount of *Streptococcus mutans*. Human saliva contains a spectrum of proteins with antimicrobial properties, among which are immunoglobulin IgA, enzymes (e.g., lysozyme, lactoferrin, and peroxidase), mucin glycoproteins, agglutinins, proline-rich proteins, and histatins. Saliva’s impact on oral bacteria has been studied from the perspective of antimicrobial activities, anti-coaggregation properties, point of adhesion, and as a carbon source^5^. The acquired enamel pellicle is derived mainly from salivary proteins and glycoproteins^6–8^ and mediates initial bacterial colonization on the tooth surface. Cheaib *et al*. demonstrated that modulation of composition in salivary proteins and glycoproteins during pellicle formation influenced subsequent adhesion of early colonizers to enamel surfaces^9^. Key receptor polysaccharides (RPS) that are important for coaggregations of streptococci and actinomyces have been previously elucidated^10^. Ito *et al*. demonstrated that a lectin which recognizes the Gal1-3GalNAc moiety, one of the known RPSs, may function as a potential anti-*S. mutans* agent^11^. It has been demonstrated that salivary protein composition differs between stimulated and unstimulated saliva^12^. The type of stimulus, such as tastants, has been known to alter salivary protein composition^13–15^. One proposed mechanism is alteration of the glandular contributions to whole-mouth saliva^12,16^. A caveat is that other factors may influence salivary secretions and composition, including age and inter-individual variations^17–20^.

An *ex vivo* bacterial overlay technique has been used to probe the binding patterns of oral bacteria to salivary proteins^21,22^. Previous studies have been limited in using either mechanically stimulated saliva or unstimulated saliva from a single subject, but not both types of saliva in comparison or from multiple subjects. Although some studies have utilized glandular saliva samples in which the contribution rate differs between stimulated and unstimulated, reports using whole saliva have been limited^23,24^. In addition, studies have investigated primary human dental plaque colonizers individually *in vitro*^25^. The present study focused on the impact of stimulated saliva on bacterial colonization in the oral cavity.

Our central hypothesis was that stimulating salivary secretion may alter the colonization of oral bacteria through changes in its protein composition, and this alteration can be observed through an *in vitro* bacterial overlay technique as well as in biofilm developed on enamel chips carried in an intraoral stent. We have previously reported a profile of microbial coaggregation partnerships during the initial phase of dental biofilm development^26^. Here we report the effect of chewing-gum-stimulated saliva on interactions between salivary proteins and bacterial binding properties. Unstimulated and stimulated whole saliva samples were collected from five individuals and analyzed with *in vitro* bacterial overlay. In addition, we conducted an *in situ* biofilm growth study using two individuals, comparing microflora on the enamel chip with or without gum chewing to provide a proof-of-concept that food-stimulated saliva influences bacterial binding in the human oral cavity.

## Results

### Saliva collection and *in vitro* bacterial overlay

**Figure 1:**
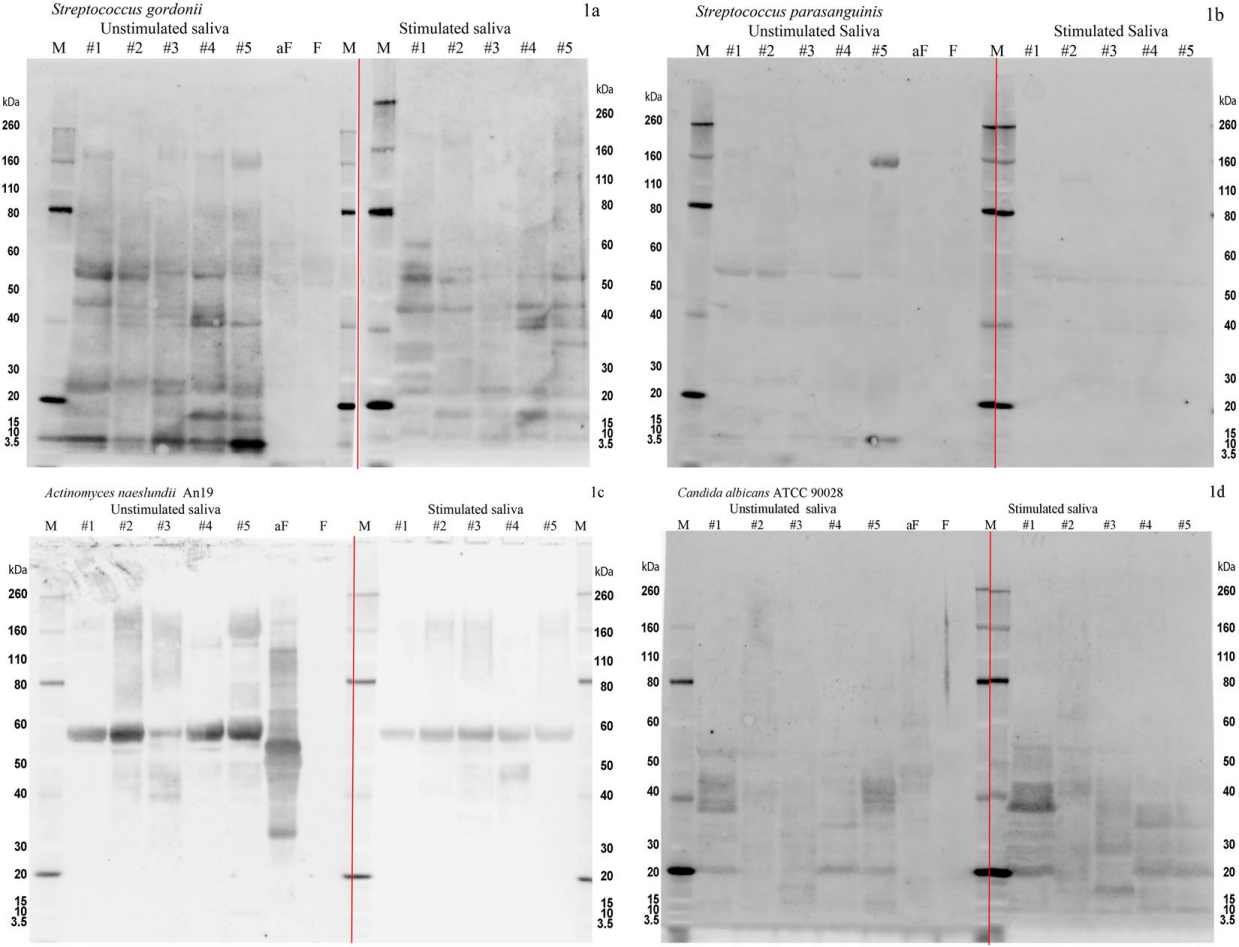
Bacterial overlay images showing binding of (**a**) *S. gordonii* ATCC 35105, (**b**) *S. parasanguinis* ATCC 15912, (**c**) *A. naeslundii* An19, and (**d**) *C. albicans* ATCC 90028, to unstimulated and stimulated saliva from 5 individuals (#1-#5). M: molecular standards (Novex^®^ sharp Prestained protein Standard). aF: asialofetuin. F: Fetuin. Exposure time for all images was 1/2 second. (**a–d**) Each represents images from two separate gels ran simultaneously. The deviding line indicates the separation between the two gels.

**Figure 2:**
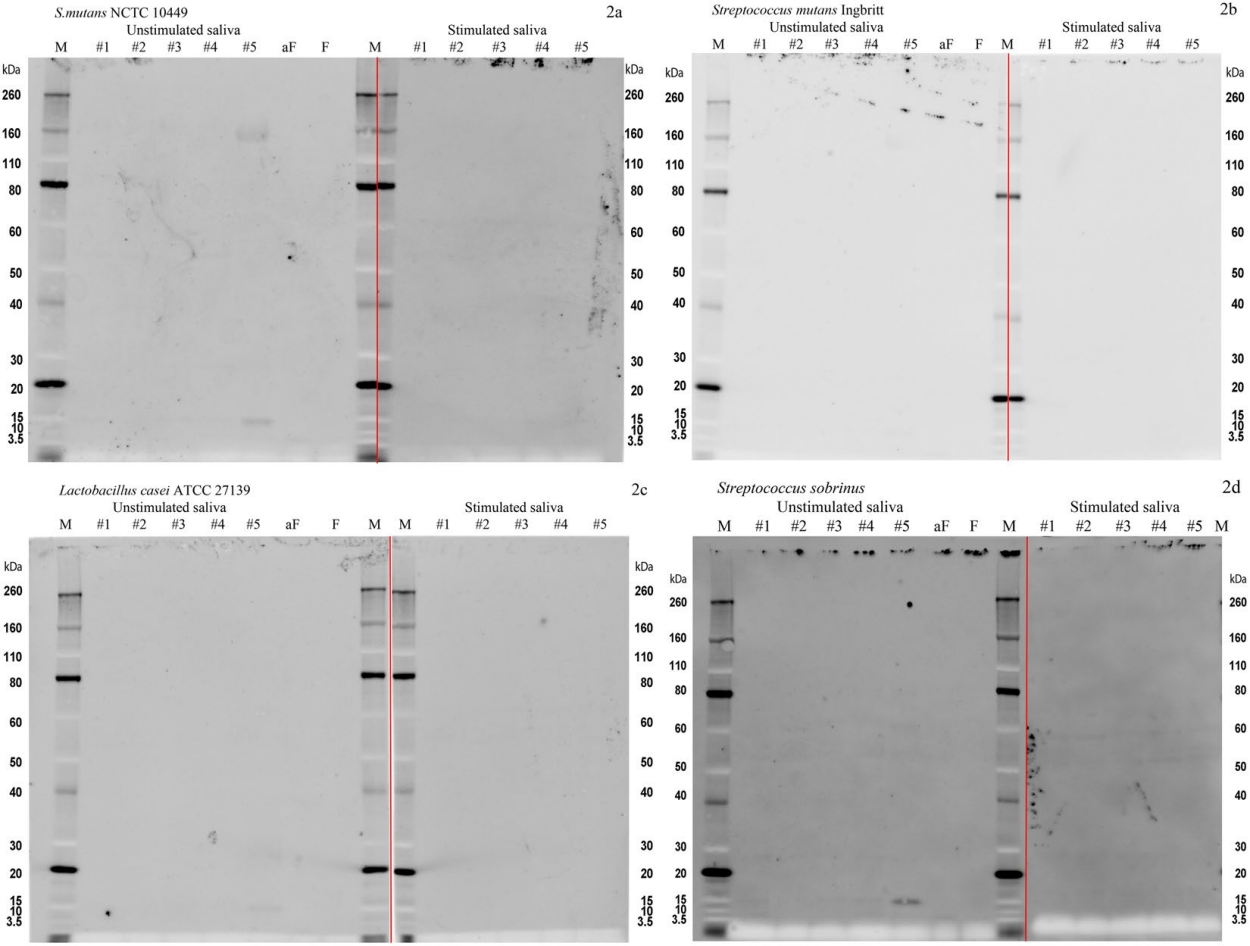
Bacterial overlay images showing binding of (**a**) *S. mutans* NCTC 10449, (**b**) *S. mutans* Ingbritt, (**c**) *L. casei* ATCC 27319, and (**d**) *S. sobrinus* ATCC 27352, to unstimulated and stimulated saliva from 5 individuals (#1-#5). M: molecular standards (Novex^®^ sharp Prestained protein Standard). aF: asialofetuin. F: Fetuin. Exposure time for all images was 1/2 second, except for *S. mutans* Ingbritt (1/4 second). (**a–d**) Each represents images from two separate gels ran simultaneously. The deviding line indicates the separation between the two gels.

The salivary flow rates were 0.416 ml/min (s.d. 0.244 ml/min) for unstimulated saliva and 1.882 ml/min (s.d. 0.703 ml/min) for the gum-base-stimulated saliva. The total protein concentrations were 1203.5 mg/ml (s.d. 276.6 mg/ml) for unstimulated saliva and 881.9 mg/ml (s.d. 198.2 mg/ml) for stimulated saliva. Analysis of data obtained from the *in vitro* bacterial overlay experiments demonstrated that test bacteria *Streptococcus gordonii*, *Streptococcus parasanguinis*, *Actinomyces naeslundii*, *Candida albicans*, and *Streptococcus sobrinus* bound to immobilized protein components of unstimulated and stimulated human saliva samples with various band intensities (Figs 1 and 2). Distinctive positive protein bands were detected at 20–30 kDa, 40–50 kDa, and at approximately 60 kDa (Figs 1 and 2). However, except for one subject (positive bands at 10 and 160 kDa), almost no binding was detected between *Streptococcus mutans* and salivary proteins. Similarly, lack of binding activity was noted for *Lactobacillus casei*. The binding pattern of *A. naeslundii* An19 to salivary proteins was distinct from those of other test bacteria. A strong band near 60 kDa was noted in both stimulated and unstimulated saliva samples (Fig. 1c).

There were clearly inter-individual differences in bacterial binding profiles among the saliva samples of the five test subjects (Fig. 1). In general, reductions in band intensities were observed in the stimulated saliva samples when compared with the unstimulated samples. For example, the protein bands at approximately 15 kDa detected in the unstimulated saliva samples were either faint (*S. gordonii*) or non-detectable (*S. parasanguinis*, *S. sobrinus*) in the stimulated saliva samples. Two control proteins, fetuin and asialofetuin, were used to monitor reproducibility of binding patterns for individual test bacteria.

### Human Oral Microbe Identification Microarray (HOMIM) analysis of *in situ* biofilm samples

**Figure 3:**
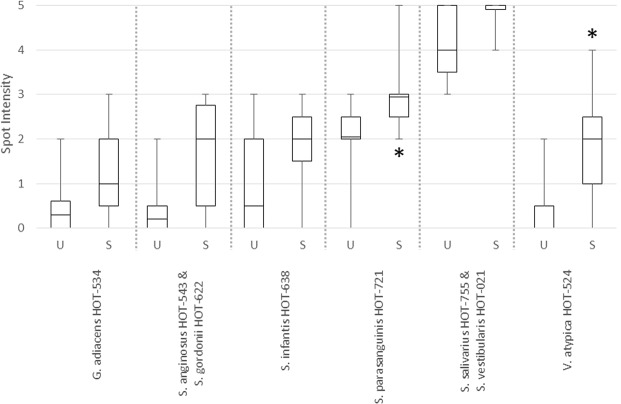
Probes indicative of the phylotypes (HOTs) that increase under salivary stimulation on enamel chips. U: unstimulated sample. S: stimulated sample. *P < 0.05.

Sixteen probes representing 26 species and 2 probes representing genus-level groups were detected in at least half of the samples collected (Table 1). Among these, 2 probes showed a significant increase under chew-stimulated conditions compared with unstimulated conditions: those corresponding to *S. parasanguinis* Human Oral Taxon (HOT)-721/411 and to *Veillonella atypica* HOT-524. Additionally, 4 probes showed a trend to increase by chewing: *Granulicatella adiacens* HOT-534, *Streptococcus infantis* HOT-638, *Streptococcus anginosus* HOT-543/*S. gordonii* HOT-622, and *Streptococcus salivarius* HOT-755/*Streptococcus vestibularis* HOT-021. Probes that showed changes between chew-stimulated and non-chew-unstimulated conditions with significant changes at *P* < 0.05 and trending changes with 0.05 < *P* < 0.15 are summarized in Fig. 3.

**Table 1:** HOMIM results: taxa included in analysis and changes by chewing.

Taxa (HOTs; listed according to probe)	Unstimulated vs Stimulated p value	Overall prevalence	Subject p value
*Actinomyces georgiae* HOT-617	N/A	0.45	
*Gemella haemolysans* HOT-626	0.389	1.00	
*Granulicatella adiacens* HOT-534	**0.090**	0.55	
*Haemophilus parainfluenzae* HOT-718	0.472	0.91	0.001
*Pseudomonas* cluster	0.799	0.64	
*Rothia dentocariosa* HOT-587/*Rothia mucilaginosa* HOT-681	0.731	1.00	0.000
*Rothia mucilaginosa* HOT-681	0.771	0.73	0.028
*Streptococcus anginosus* HOT-543/*Streptococcus gordonii* HOT-622	**0.057**	0.55	0.000
*Streptococcus australis* HOT-073	0.567	1.00	
*Streptococcus* cluster	0.499	0.73	0.001
*Streptococcus constellatus* HOT-576/*Streptococcus intermedius* HOT-644	0.782	0.55	
*Streptococcus downei* HOT-594	0.443	0.55	
*Streptococcus infantis* HOT-638/*Streptococcus* sp. HOT-065	**0.104**	0.91	
*Streptococcus mitis* bv2 HOT-398/*Streptococcus* sp. HOT-069	0.310	0.64	0.004
*Streptococcus oralis* HOT-707/*Streptococcus* sp. HOT-064	0.539	1.00	
*S. parasanguinis* I HOT-721/*S. parasanguinis* II HOT-411/*S*. sp. HOT-057	**0.045**	1.00	0.002
*S. salivarius* HOT-755/*S. vestibularis* HOT-021/*Streptococcus* sp. HOT-067	**0.138**	1.00	
*Veillonella atypica* HOT-524	**0.016**	0.82	

Major probes indicate species (HOTs) which showed prevalence of 0.5 in the enamel chip samples obtained with unstimulated or stimulated saliva. P value was not calculated where the prevalence was < 0.5. Numbers in bold indicate the trend (p < 0.15); numbers in bold and underlined indicate the significant change (p < 0.05).

### Imaging of biofilm *in situ* on the chips

**Figure 4:**
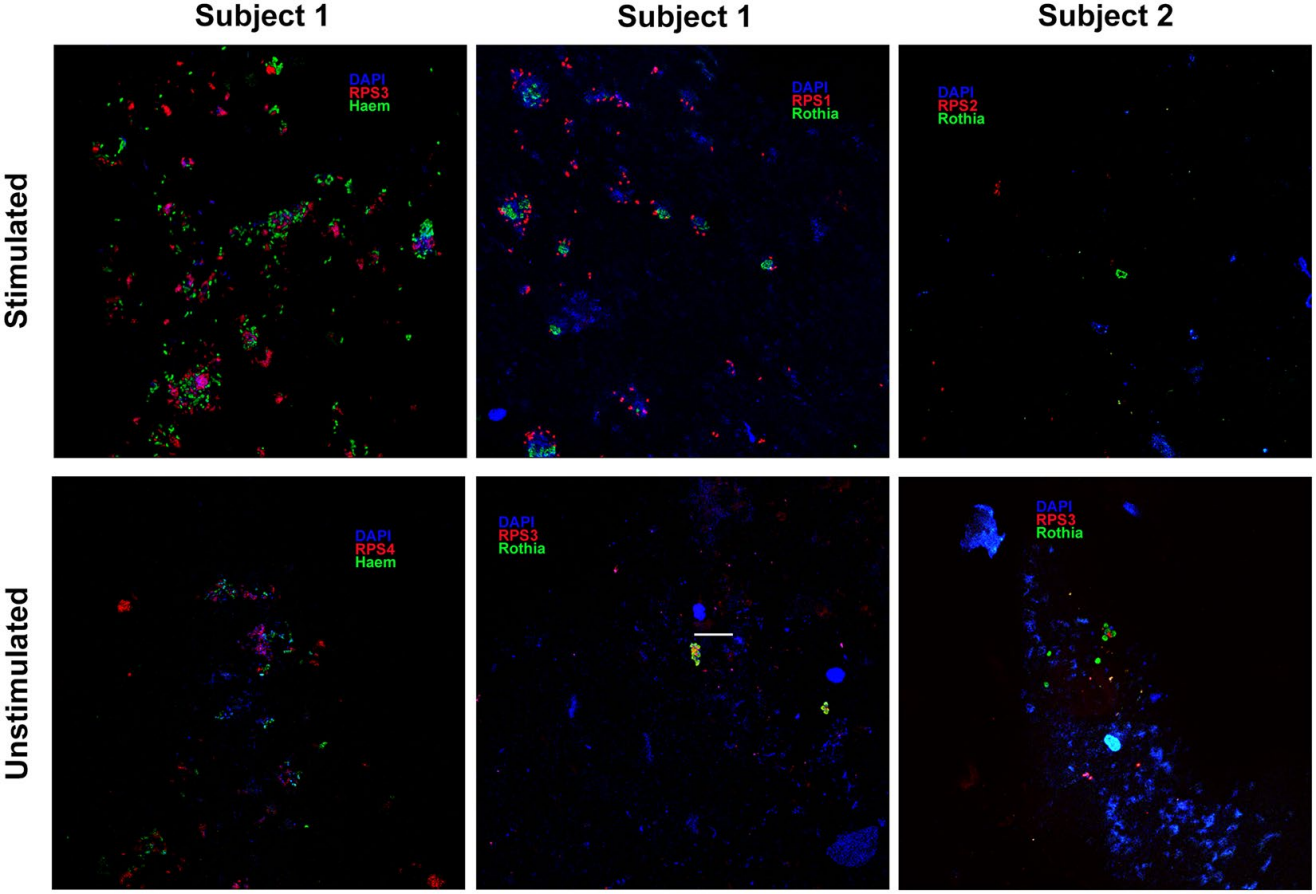
Representative images of biofilms stained with a mixture of fluorescently labeled antibodies against different RPS serotypes, against whole cells of *Rothia mucilaginosa*, and against whole cells of *Haemophilus parainfluenzae*. Note that colors for RPS, *Rothia*, and *Haemophilus* are not identical across the images. Clusters composed of several cell types are visible in each subject. Cells reactive with antibody against RPS serotypes 3 and 4 were seen in high numbers in Subject 1. RPS serotype 4-reactive cells were absent in Subject 2. Further, anti-*Haemophilus-*reactive cells were not seen on chips from “no chewing” experiments in Subject 2, which likely reflects the low overall biomass. Scale bar = 20 µm and applies to all panels.

Images of antibody-stained biofilms on chips worn while subjects chewed are shown in Fig. 4 and in Supplementary Figs 1–3. Single cells made up the bulk of the biofilm biomass and, when confluent biofilm was present, it was unevenly distributed across the chip. Biomass on chips from Subject 2 was visibly lower compared with that on chips from Subject 1. Cells reactive with antibody against RPS serotypes 3 and 4 were present in high numbers in Subject 1 but were rare in Subject 2. Cells reactive with antibodies against RPS serotype 2 were seen in both individuals in low abundance. Cells reactive with antibody against RPS serotype H1 were rare in both individuals. Anti-*Haemophilus*- and anti-*Rothia*-reactive cells occurred in both subjects. Cells reactive with antibodies against *Actinomyces* strains were not found in either subject. Importantly, mixed-species colonies consisting of a few cells were easily found. In summary, *Rothia, Haemophilus*, and RPS-bearing streptococci occurred in both subjects, and interindividual differences were seen in RPS serotype abundance. However, intra-individual chewing-dependent differences in the types of RPS-reactive cells or in the amount of biomass were not apparent.

## Discussion

There was a consistency in binding patterns across saliva samples from multiple subjects, although some protein bands exhibited binding with higher intensity in some subjects than in others. In all strains tested except for *A. naeslundii*, the binding to proteins with MW above 80 kDa was diminished with stimulated saliva compared with unstimulated saliva. The salivary proteins in this molecular-weight range are often assigned as proline-rich proteins (PRPs) and mucin MUC7. Bands around 40 kDa, often assigned as the fragments of PRPs, were not affected by the type of saliva for all strains. Bands around 55 kDa, typically assigned to salivary amylase, showed decreased intensity with stimulated saliva for *S. gordonii* and *S. parasanguinis;* however, no change was observed for the other strains. No band showed a consistent increase in its intensity with stimulated saliva. Proteins and peptides with MW range between 5 and 40 kDa are comprised of statherin, histatins, cystatin, and PRPs^27–29^. These molecules are considered to play an important role in initial colonization. Two strains, *S. gordonii* and *S. parasanguinis*, showed less intense binding to the bands in this range when tested with stimulated saliva. Considering that stimulated saliva has a lower total protein concentration than does unstimulated saliva, this suggests that the key binding proteins differ between stimulated and unstimulated saliva^12^. Stimulated saliva is known to have a higher contribution from the parotid gland, and its flow rate remains elevated as long as masticatory stimulation continues^13,30,31^. Our results showed an overall agreement with results from a previous study of the interaction of *S. gordonii* and salivary proteins^25^. This work exposed *S. gordonii* to labeled salivary proteins, and the bacteria-bound proteins were eluted from the cell surface, separated by SDS-PAGE, and identified. They demonstrated strong positive protein bands between 50 and 60 kDa (putatively salivary amylase) and positive but faint bands between 20 and 30 kDa (putatively IgA κ and λ chains) and between 10 and 15 kDa (putatively lysozyme or cystatin)^32^. Our *in vitro* binding method involved overlaying labeled bacteria on SDS-PAGE-separated salivary proteins. Our results were similar to those previously reported, except that we observed strong binding to proteins at 160 to 170 kDa, probably putatively salivary mucin MUC7, which was not reported^25^. Among the strains of *S. mutans* tested, Ingbritt and NCTC 10449 did not demonstrate binding to the salivary proteins. This could explain why *S. mutans* species are not usually found as initial colonizers of dental biofilm. Similarly, we noted negative or weak binding with the other two cariogenic bacteria, *L. casei* and *S. sobrinus*. Our results suggested that there are differences in bacterial binding profiles among selected supragingival biofilm bacteria (Fig. 1), and caries-associated taxa (Fig. 2) were detected. The reduced number of bands in *in vitro* binding to stimulated saliva showed that chewing may affect how oral bacteria bind to enamel surfaces and provide a more competitive environment for binding scaffolds for bacteria-protein interactions when saliva is stimulated by chewing.

Between the two *in situ* analyses, HOMIM provides a non-selective semi-quantitative picture of the total bacterial community. Table 1 lists HOTs with threshold values of > 0 in either chewing or non-chewing samples. It is noteworthy that only 2 HOTs showed a statistically significant change when subjects chewed. An additional 4 probes representative of 6 HOTs showed a trend in changes with subjects chewing. These results suggest that chewing does not have a broad common effect on community composition across the two individuals. We understand the limitation in drawing conclusions based on an *in situ* study with only two subjects. Future studies with increased numbers of subjects are warranted.

All 6 probes in Fig. 3 increased their spot intensity with stimulated saliva compared with those formed under unstimulated saliva. These probes cover 8 HOTs, including *S. gordonii* and *S. parasanguinis*, which have been previously identified as initial colonizers of biofilm. *V. atypica* is a known coaggregation partner with *S. gordonii* and is also associated with *S. parasanguinis* in biofilm formation^33,34^. *Veillonellae* are also known to vary in phenotype during plaque accumulation^35^. Five of these 8 HOTs have been associated with oral health: *Gr. adiacens*, *S. gordonii*, *S. parasanguinis*, *S. salivarius* and *V. atypica*^34^.

The major HOTs found in chewing and non-chewing biofilms were much the same between subjects and visits. All organisms except *Pseudomonas* are frequently reported as typical and major components of supragingival plaque. The presence of *Rothia, Streptococcus*, and *Veillonella* in chip biofilm is also in agreement with their reported roles in initial biofilm formation^1^. In this work, we also observed high prevalence of the microorganisms previously associated with soft-tissue surfaces, including *Pseudomonas* spp. and *Streptococcus australis*. The latter is a relatively common, newly classified species (ranked as the 39th most frequent organism in the HOMD database), which has only rarely been discussed in previous HOMIM studies or in culture studies. The lack of culture study data on *S. australis* could likely reflect the fact that the species had not been taxonomically separated from other streptococcal species until 2001. It is noteworthy that the importance of this soft-tissue-associated bacterium did not increase after chewing in either sample. One consideration is that differences between biofilms of chewing-stimulated samples vs unstimulated samples may reflect dislodgement of bacteria from the oral epithelium rather than selective attachment related to the presence/absence of particular proteins in saliva. While it is difficult to eliminate the mechanical factors in these *in situ* studies, few differences were noted between biofilms on chips from stimulated-saliva volunteers and those on chips from unstimulated-saliva volunteers. Limited numbers of epithelial cells were detected on chips after their removal from the mouth, suggesting that mechanical factors play a small role (data not shown).

A parallel study on these same subjects reported a broad overview of HOMIM results from the “no chewing” arm of these experiments^26^. Those results demonstrated the prominence of *Rothia* spp. and *Haemophilus parainfluenzae* in the biofilms of both subjects, as well as a high degree of interaction between these species and other cells in the biofilm. Similar intimate cell-cell interactions of *Rothia* and *Haemophilus* with other cells were clear in the present study, independent of chewing. Also important is the absence of anti-*Actinomyces*-reactive cells in the biofilms; this was clearly corroborated by the low *Actinomyces* HOMIM probe values in the present study as well as in the previous study^26^. Thus, while an *Actinomyces* strain showed chewing-dependent differences in salivary binding profile (Figs 1 and 2), it is unlikely that such a difference could be detected in the imaging or the HOMIM data from these subjects. *Actinomyces* spp. are known to be important to early biofilm based on molecular and imaging analyses^36,37^, but the broad range of phenotypes makes it difficult to correlate the species- and strain-specific data acquired through *in vitro* studies (salivary binding) with the phenotype-independent data acquired by antibody-based imaging and HOMIM analyses.

Microscopic data collected in the present study are qualitative in nature and can be related to those reported in an earlier study^25^ with respect to correspondence of anti-RPS-reactive cells with RPS-bearing streptococcal isolates in these same subjects; at least one RPS-bearing isolate from both subjects corresponded to the presence of the appropriate anti-RPS-labeled cells in the biofilm. Therefore, while RPS antibodies used together with *in situ* imaging could be a good indicator of chewing-dependent effects on biofilm community composition, such effects were not obvious here. Also, RPS antibodies do not correlate with detailed taxonomic assignments (e.g., strains within the same species of streptococci can vary in the presence and serotype of RPS), and no HOMIM probe exists for certain prominent streptococcal species known to bear RPS^26^. With regard to salivary-binding profiles, it is clear that the streptococcal species showed no clear chewing-dependent differences; this supports the broader perspective gained by analysis of the imaging data. However, subject-specific differences in biofilm composition are evident in the imaging data of the present study, as well as in HOMIM data of the previous study^26^. Thus, in the present study, only inter-subject differences were obvious within the imaging datasets, and these were not affected by chewing.

In summary, the overlay results demonstrated binding to salivary proteins by early-colonizing species, while other bacteria important in caries did not bind well. The results also showed fewer proteins available for binding with stimulated saliva. Analysis of the HOMIM data indicates the consistent and prominent presence in all samples of commonly occurring tooth-surface bacteria. These include *Gemella haemolysans*, *Rothia* spp., *Haemophilus parainfluenzae*, and many different *Streptococcus* spp. Three common tooth isolates – *Rothia mucilaginosa*, *S. parasanguinis*, and *V. atypica* – showed an increase after subjects chewed. Another common oral isolate, *S. gordonii*, showed a trend to increase when subjects chewed. These initial colonizers are associated with health. Immunofluorescence with anti-RPS antibody provided support for the HOMIM data. A major difference was observed in the bacteria-protein interactions between subjects. Not only did the binding pattern of a type strain show clear differences between subjects, but also microscopy of *in situ* biofilms showed clear differences in the presence of some anti-RPS-reactive cells. This demonstrates a component of inter-individual variability not acquired with a single approach. Chewing appeared to affect bacterial binding profiles during very early colonization (4 hours) on at least some of the known early colonizers. The response of stimulated saliva to chewing seems to be individual-specific, and therefore further research should focus on functional analysis of bacteria or the impact on co-aggregation/co-adhesion.

## Materials and Method

### Unstimulated and stimulated saliva collection

Five adult non-smokers (three male and two female 18 to 65 years old) who did not chew gum regularly and had no reported health condition or medication intake were enrolled in the study. Their resting unstimulated saliva was collected first by the drooling method^38^. Then their stimulated saliva was collected when they spit into a 50-ml centrifuge tube for 10 minutes while chewing a 1-g piece of unflavored unsweetened gum base (Mars-Wrigley Confectionery, Chicago, IL, USA). Samples were placed on ice immediately upon collection, and phenylmethylsulfonyl fluoride was added at a final concentration of 1 μM. Saliva samples were clarified by centrifugation at 12,000 × *g* for 10 minutes at 4 °C, and the supernatant was stored at −80 °C until further analysis. Total protein was measured by the Lowry method (Sigma-Aldrich, St. Louis, MO, USA). All experimental protocols were preapproved by the University of Illinois at Chicago (UIC) Institutional Review Board (IRB #2006-0916). All parcipants provided informed consent prior to the initiation of the study procedures.

### Test microorganisms and growth

*Actinomyces naeslundii* (strain An19), *Streptococcus gordonii* ATCC 35105, *Streptococcus mutans* NCTC 10449, *S. mutans* subsp. Ingbritt, *Streptococcus sobrinus* ATCC 27352, and *Streptococcus parasanguinis* ATCC 15912 were grown in Brain Heart Infusion broth (BHI, Bacto Laboratories, Mt Pritchard, NSW, Australia). *Lactobacillus casei* ATCC 27319 was grown in the MRS medium (BD Difco, Franklin Lakes, NJ, USA). *Candida albicans* ATCC 90028 was grown in RPMI 1640 medium (Sigma-Aldrich). All test organisms for the overlay study were grown in 10-ml tubes of broth without shaking at 37 °C in a 5% CO_2_ incubator.

### *In vitro* bacterial binding to salivary proteins

Bacterial binding to salivary proteins was studied by an overlay method^21^ in which saliva samples (20 μg protein per lane) were separated by SDS-PAGE on a 4–12% Bis-Tris gel (NuPAGE^TM^, Invitrogen, Carlsbad, CA, USA). Proteins were then electro-blotted onto a PVDF membrane (BIO-RAD, Hercules, CA, USA) and overlaid with 9.0 ml of FITC-labeled bacterial cells (5 × 10^8^ cells/ml) suspended in block solution (TBS buffer with 5% BSA, 1 mM CaCl_2_, 1 mM MgCl_2_). The bacteria-protein binding patterns were identified as fluorescent bands under UV epi-illumination (ImageQuant LAS4000; ex 494 nm/em 518 nm). Two standard proteins, namely, fetuin and asialofetuin (Sigma-Aldrich), were loaded onto separate lanes in each of the SDS-PAGE gels as controls.

### *In situ* biofilm growth, community composition analysis, and biofilm imaging

Fourteen adults (18 to 64 years old) were recruited for an initial screening. Inclusion criteria were: healthy (self-reported medical history) adults at least 18 years old and having good oral health as determined by a dentist’s examination. Exclusion criteria were: active caries, oral cancer, or more than mild gingivitis; history of medical conditions affecting salivary flow; a history of immunosuppressant therapy; history of cardiac, kidney, liver, or lung disorders; use of tobacco within the last year: use of antibiotics within the preceding four months; use of medications or radiation therapy thought to affect salivary flow, such as head/neck radiation, diuretics, or nitrates; current steroid therapy (other than topical) within the preceding 30 days; auto-immune or immune diseases such as ulcerative colitis or systemic lupus erythematosus; active dental caries; moderate to severe gingivitis; and periodontal probing depths of >3 mm on two or more molars and premolars. These criteria resulted in the recruitment of only those subjects with excellent oral health. All studies were carried out in accordance with approved National Institute of Health (NIH) guidelines including prior review by the NIH intramural Institutional Review Board, and in accordance with the standards indicated by the Declaration of Helsinki. All study participants provided informed consent prior to the initiation of any study procedures. Two male subjects (hereafter referred to as Subject 1 and Subject 2) were selected to continue with the study based on the above criteria, and after confirmation that biofilm on intra-orally carried retrievable enamel chips contained bacteria that stained with anti-RPS antibodies (see below).

Enamel chips (3 mm × 3 mm) were cut from extracted third molars that had been thoroughly rinsed and air-dried. The chips were then sonicated in a low-power bath (model 1510, Branson, Danbury, CT, USA) for 10 minutes. Efficacy of the cleaning procedure was demonstrated by epifluorescence microscopy; a chip that had been stained with DAPI bore no bacterial or cellular debris. After sterilization by ethylene oxide, three chips each were affixed to both the left and the right buccal sides of a full-mandibular stent by means of dental wax. The stent was then worn by the participants for 4 hours, after which the enamel chips were removed and investigated by confocal microscopy and microbial community analysis (HOMIM). In a cross-over design, both subjects reported for 6 visits, 3 visits in which gum was chewed, and 3 in which no gum-chewing took place. For chewing experiments, subjects chewed a piece of chewing gum (2.7 g; Wrigley Extra^®^) for 10 minutes without the stent in place, immediately after which the stent was inserted. After 4 hours, the stent was removed, and DNA was extracted from the biofilm as described previously^26^. Briefly, the enamel chips were gently dipped in sterile water three times, then sonicated. DNA was extracted from the sonicate by means of a DNAeasy Blood and Tissue kit (QIAGEN, Valencia, CA, USA), then sent to The Forsyth Institute (Boston, MA, USA) for biofilm community composition analysis by HOMIM^39^. In this procedure, extracted sample DNA is amplified with a universal 16S primer, then resulting amplicons are fluorescently labeled and hybridized to the array that bears short DNA sequences (probes) corresponding to 16S ribosomal gene sequences of taxa (Human Oral Taxon; HOTs) within the Human Oral Microbiome Database (HOMD; http://expanded.homd.org/). Fluorescent signal intensity (values of 0–5) is read for each spot, which corresponds to the amount of a particular phylotype (or, in cases of multiply-reactive probes, to several phylotypes) in the sample.

In addition, the biofilm on at least one chip from each experiment was stained with a mixture of fluorescently labeled primary antibodies raised against purified RPS and against whole cells of *Actinomyces*, *Rothia*, and *Haemophilus* strains, as well as with DAPI (to reveal antibody-unreactive cells)^26^. The biofilms were visualized by means of a 0.9NA 60x water-immersible (dipping) lens mounted on a Zeiss LSM 710 confocal microscope.

### Statistical analysis

HOMIM results are reported as probe abundance with values ranging from 0 (absent) to 5 (abundant). The rate at which each probe was detected from all visits was defined as prevalence. Only probes with a value > 0 in at least half the visits of either ‘chew’ or ‘non-chew’ were used for analysis, i.e., prevalence >0.5; these were defined as the major probes. Analyses were performed on the absolute intensity of HOMIM data (0–5) by non-metric MDS (MultiDimensional Scaling) and a non-parametric ANOVA with PAST 3.08 (Paleontological Museum, University of Oslo, Oslo, Norway). For the change in individual probe values, the Mann-Whitney test was performed with IBM SPSS 23.0 (IBM Corp., Armonk, NY, USA). A *P* value of < 0.05 was considered statistically significant after the Benjamini-Hochberg correction^40^.

## Supplementary information

supplemental Data
