# Supplementary material for: Effect of mechanically stimulated saliva on initial human dental biofilm formation
Source: Sci Rep. 2019 Aug 14;9:11805. doi: 10.1038/s41598-019-48211-3 (PMC6694102; doi:10.1038/s41598-019-48211-3)
Supplement: Supplementary file 1 — supplemental Data [file 41598_2019_48211_MOESM1_ESM.pdf]

Supplementary information for:

Effect of mechanically stimulated saliva on initial human dental biofilm formation.

Taichi Inui, Robert J. Palmer, Jr., Nehal Shah, Wei Li, John O. Cisar & Christine D. Wu

Supplementary Figure 1.
Antibody-stained cells of low-abundance or absent in Subject 1. All images are from "chewing" samples. Scale bar = 20 μm. Right images are 3x zoom of left images.

Upper row: Antibody-unreactive cells (DAPI-stained, blue) are abundant, as are anti-RPS4-stained cells (green). In contrast, anti-RPS2-stained cells (red) are few and anti-*Actinomyces*-stained cells (would be magenta) are absent.

Lower row. Antibody-unreactive cells (DAPI-stained, blue) are abundant. Anti-*Actinomyces*-stained cells (would be green) are absent. Anti-RPS3-stained cells (red) are present. A single anti-H1-stained cell (magenta) is present (arrow).

Supplementary Figure 2.
Antibody-stained cells of low-abundance or absent in Subject 2. All images are from "chewing" samples. Scale bar = 20 μm. Right images are 3x zoom of left images.

Upper row: Antibody-unreactive cells (DAPI-stained, blue, much DAPI-stained material is epithelial cell nuclei) are sparse, reflecting lower biomass overall than for Subject 1. Anti-*Haemophilus*-stained cells (would be green) are absent. Anti-RPS1-stained cells (red) are present. Two anti-H1-stained cells (magenta) are visible (arrows).

Lower row: Antibody-unreactive cells (DAPI-stained, blue, much DAPI-stained material is epithelial cell nuclei) are sparse, reflecting lower biomass overall than for Subject 1. Anti-RPS4-stained cells (would be green) are absent. Four anti-Haemophilus-stained cells (red) are present. Anti-H1-stained cells (would be magenta) are absent.

Supplementary Figure 3
Antibody-stained cells of low-abundance or absent in Subject 2. All images are from "chewing" samples. Scale bar = 20 μm. Right images are 3x zoom of left images.

Upper row: Antibody-unreactive cells (DAPI-stained, blue) are present. Anti-Haemophilus-stained cells (would be green) are absent. Anti-RPS1-stained cells (red) are present. Anti-Actinomyces-stained cells (would be magenta) are absent.

Lower row: Antibody-unreactive cells (DAPI-stained, blue, much DAPI-stained material is epithelial cell nuclei) are sparse, reflecting lower biomass overall than for Subject 1. Anti-Actinomyces-stained cells (would be green) are absent. Anti-RPS3-stained cells (red) are few.

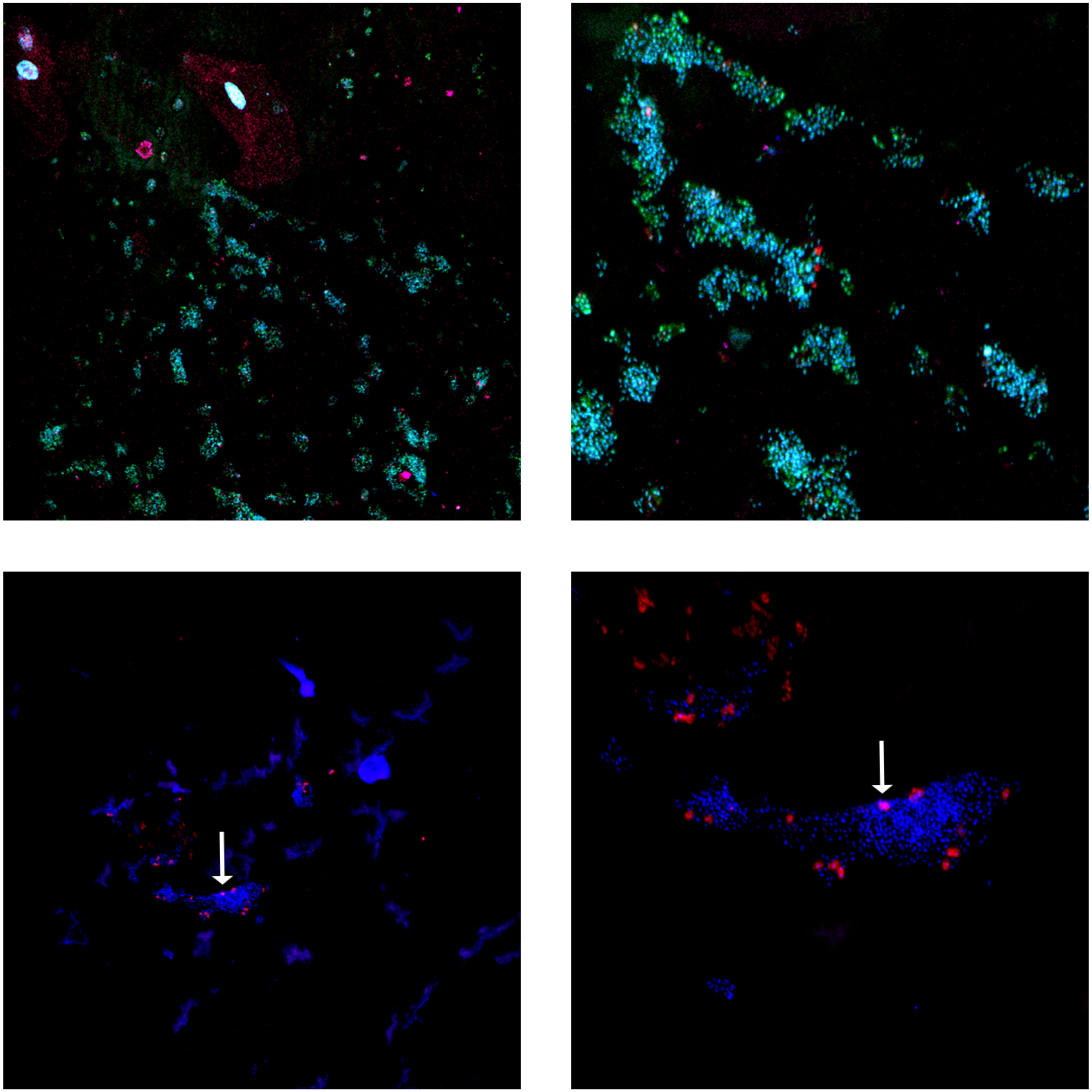

Supplementary Figure 1

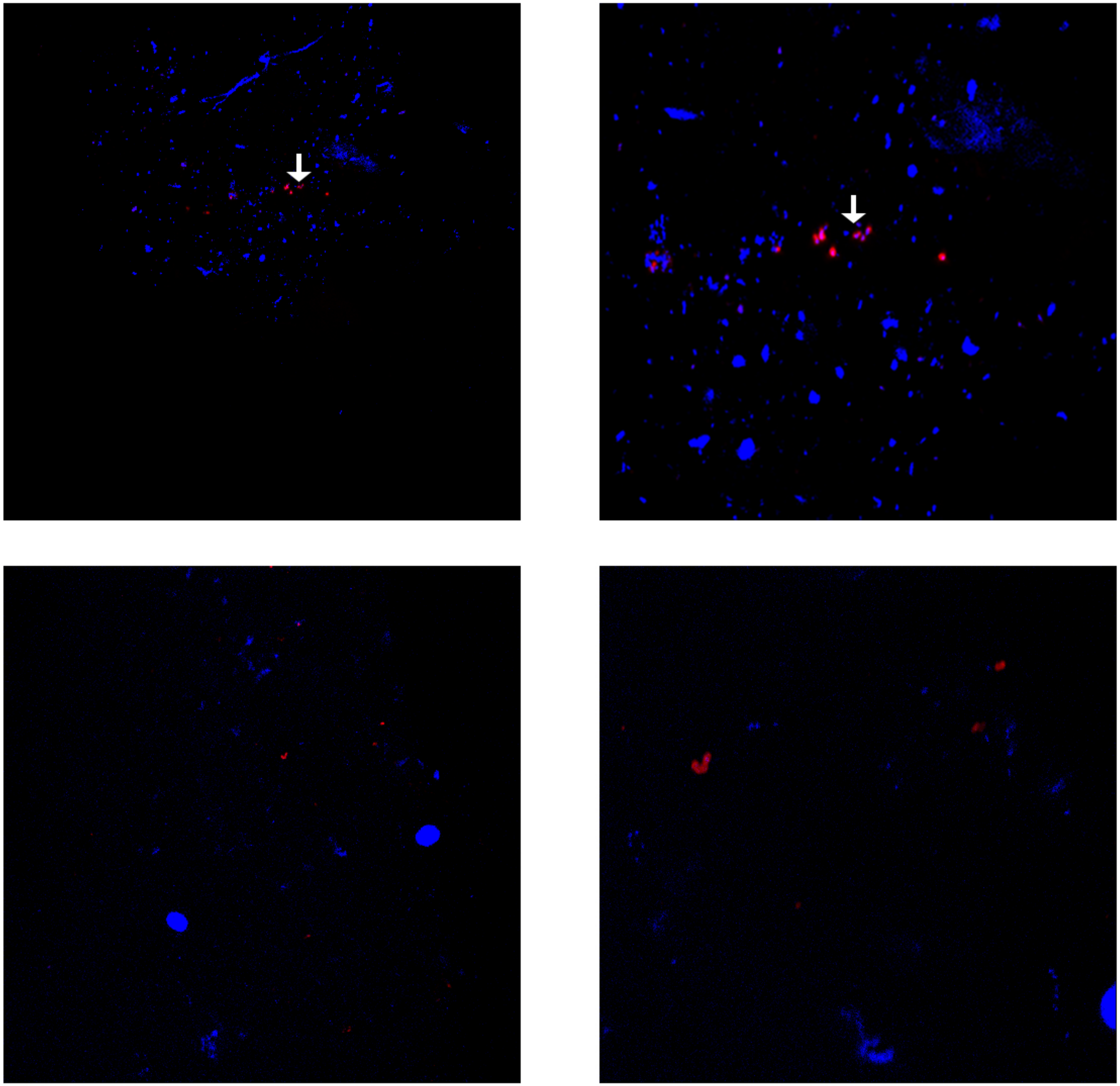

Supplementary Figure 2

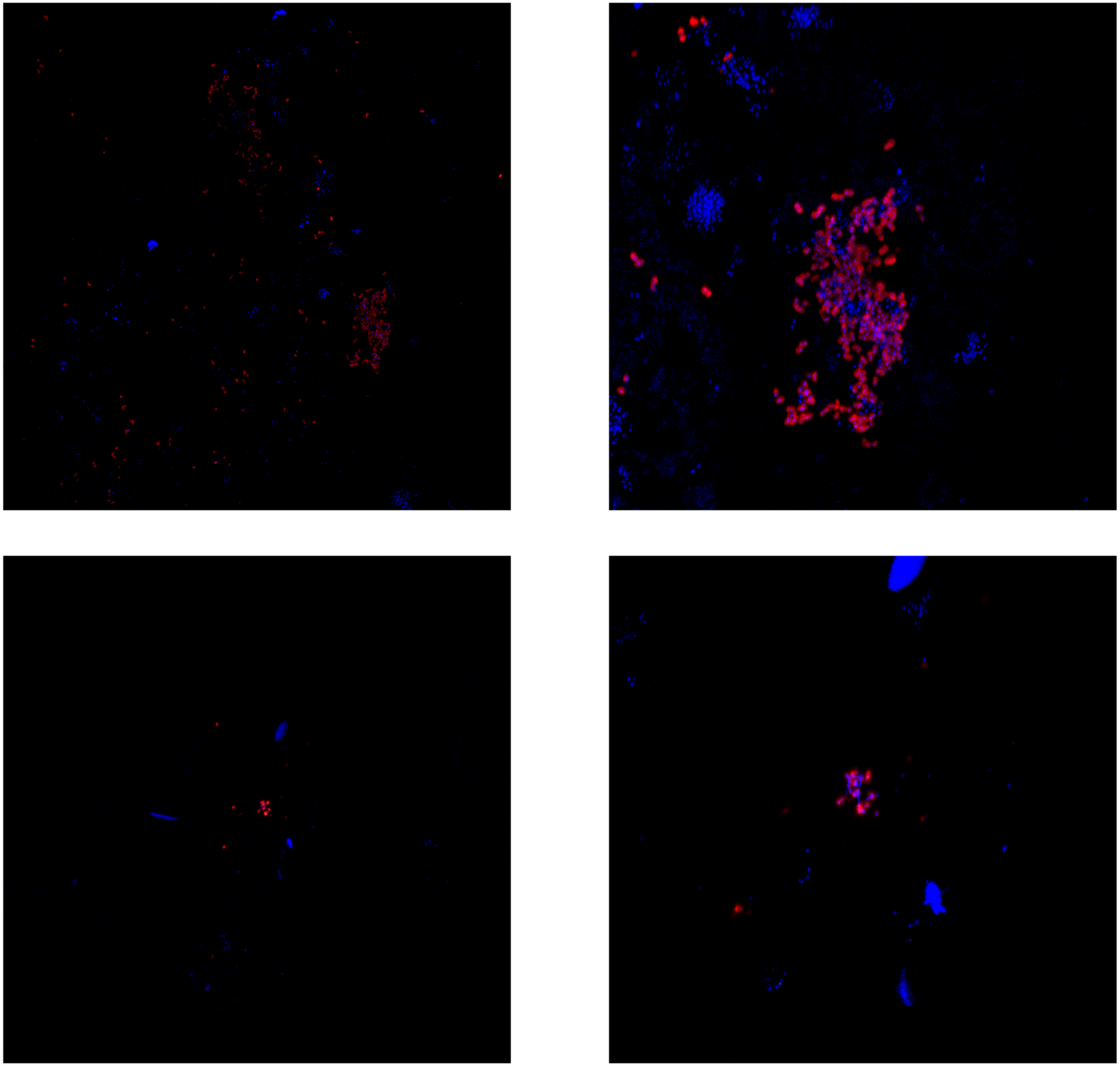

Supplementary Figure 3
